# The Guardian of the Genome Revisited: p53 Downregulates Genes Required for Telomere Maintenance, DNA Repair, and Centromere Structure

**DOI:** 10.3390/cancers10050135

**Published:** 2018-05-06

**Authors:** Eléonore Toufektchan, Franck Toledo

**Affiliations:** 1 Genetics of Tumour Suppression, Equipe Labellisée Ligue, Institut Curie, Centre de recherche, 26 rue d’Ulm, 75248 Paris Cedex 05, France; Eleonore.toufektchan@curie.fr; 2 CNRS UMR 3244, Paris 75005, France; 3 Sorbonne Université, Paris 75005, France; 4 PSL Research University, Paris 75005, France

**Keywords:** p53, telomeres, centromeres, DNA repair, bone marrow failure syndromes, aging, tumor suppression

## Abstract

The p53 protein has been extensively studied for its capacity to prevent proliferation of cells with a damaged genome. Surprisingly, however, our recent analysis of mice expressing a hyperactive mutant p53 that lacks the C-terminal domain revealed that increased p53 activity may alter genome maintenance. We showed that p53 downregulates genes essential for telomere metabolism, DNA repair, and centromere structure and that a sustained p53 activity leads to phenotypic traits associated with dyskeratosis congenita and Fanconi anemia. This downregulation is largely conserved in human cells, which suggests that our findings could be relevant to better understand processes involved in bone marrow failure as well as aging and tumor suppression.

## 1. Introduction

First identified in complex with the SV40 tumor-virus oncoprotein [1,2,3,4], p53 was initially described as an oncogene [4,5]. However, further investigations in the 1980s reclassified the protein as a major tumor suppressor. The *TP53* gene is mutated in about half of human cancers [6,7,8] and the inheritance of a mutant *TP53* allele can lead to the Li-Fraumeni syndrome of cancer predisposition characterized by the development of sarcomas and other cancers before 45 years of age [9]. Moreover, p53^−/−^ mice, knocked out for the *Trp53* gene, develop cancers (mainly lymphomas and sarcomas) with 100% penetrance [10,11,12,13].

Understanding p53 regulation and functions has been a major research aim since the discovery of this protein. p53 is now known to be post-translationally modified, stabilized, and activated in response to cellular stress such as DNA damage [14,15], oncogene expression [16], or ribosome dysfunctions [17,18,19] and to activate the transcription of an important number of direct target genes mainly implicated in cell cycle arrest (*CDKN1A*/*p21*) [20], DNA repair (*DDB2*, *XPC*, *GADD45A*) [21,22,23], apoptosis (*BAX*, *BBC3/PUMA*, *NOXA*) [24], and senescence (*CDKN1A*/*p21*, *PAI1*, *PML*) [25,26]. p53 is also able to enhance metabolic changes and antioxidant responses [27,28,29]. By maintaining genome integrity and preventing the proliferation of cells with damaged DNA, p53 acts as “the guardian of the genome” and prevents tumorigenesis [30]. 

p53 is a protein of 393 residues (in humans) composed of five proposed domains among which the core DNA binding domain (residues 100–300) is essential for the specific binding of p53 to response elements in the promoters of its target genes. The missense mutations most frequently found in human cancers (known as hot-spot mutations) are localized within this core domain [31]. These mutations mainly act by disrupting p53 capacity to bind DNA or by altering the folding of the domain. Therefore, this prevents p53 from performing its transcription factor activity [32]. Additionally, p53 can bind DNA in a non-sequence specific manner through its C-terminal domain (residues 363–393) [33]. The lysine-rich C-terminal domain is also recognized as a regulatory domain, which regulates p53 activity and stability through multiple post-translational modifications. However, its impact remained controversial for many years due to contradictory data obtained from in vitro approaches or studies relying on the transfection of p53 expression vectors (see References [34,35,36] for recent reviews and discussion).

In recent years, mouse models with targeted p53 mutations were found to be more reliable than transfection approaches when studying p53 regulation [31]. Moreover, such mouse models can reveal unsuspected functions of p53. In this review, we discuss the insights gained from our recent analyses of mice expressing p53^∆31^, which is a mutant protein that lacks the C-terminal domain [36]. This mouse model exhibited increased p53 activity, which demonstrates that the p53 C-terminus plays a negative regulatory role on the protein. Surprisingly, it also revealed that increased p53 activity may alter the genome through the downregulation of genes involved in telomere maintenance, DNA repair, and centromere structure, and lead to the development of phenotypic traits associated with bone marrow failure syndromes.

## 2. Removing the p53 C-Terminal Domain Leads to p53 Activation

The mouse model p53^∆31^ expresses a mutant p53 protein truncated of its last 31 amino acids, which corresponded to the entire C-terminal domain. This mutation removes many sites of p53 post-translational modifications among which lysine residues that can be ubiquitinated or acetylated to impact p53 stability and activity. The mutant p53^∆31^ appeared more stable than the wild-type counterpart and could be further stabilized in response to stress. Although the truncated protein did not bind DNA more efficiently, its increased stability likely contributed to an overall increase in activity, which was demonstrated by the increased transactivation of well-known p53 target genes (*Cdkn1a/p21*, *Mdm2*, *Bbc3/Puma*…) in mouse embryonic fibroblasts (MEFs) unstressed or in response to DNA damage, as well as the premature senescence of MEFs or the increased apoptosis of thymocytes. Furthermore, the p53^Δ31/Δ31^ homozygous mice exhibited traits previously reported in several mouse models with increased p53 activity [37,38,39,40,41] such as short stature, skin hyperpigmentation, cerebellar hypoplasia, testicular atrophy, heart hypertrophy, and an aplastic anemia generally lethal two to six weeks after birth [36]. This mouse model, therefore, provided evidence that deleting the p53 C-terminal domain leads to p53 activation in many different cell types and tissues such as fibroblasts, thymocytes, keratinocytes, testis, cerebellum, and bone marrow. Another mouse p53 mutant, truncated of the last 24 amino acids and referred to as p53^ΔCTD^, was later reported [42]. Consistent with our findings, p53^ΔCTD/ΔCTD^ mice were smaller than their littermates, suffered from hematopoietic failure and cerebellum hypoplasia, and had an increased p53 activity observed in their bone marrow, thymus, and spleen. A decreased p53 activity was detected in their liver, which suggested tissue-specific effects of the p53^ΔCTD^ mutation which remain to be elucidated [42]. 

Several other mouse models support the conclusion that the C-terminus has an overall negative regulatory role on p53 activity. p53^7KR/7KR^ mice resulting from the targeted mutations of 7 C-terminal lysine residues into arginines were generated to test the impact of lysine post-translational modifications on p53 function. A modest increase in p53 activity was first observed in p53^7KR/7KR^ thymocytes after γ-irradiation or in p53^7KR/7KR^ fibroblasts after culture stress [43] and p53^7KR/7KR^ mice were shown to be extremely radiosensitive due to an increased p53 activity in bone marrow cells [44]. Furthermore, p53^KQ/KQ^ mice resulting from the targeted mutations of the same 7 C-terminal lysines into glutamine residues were generated to mimic constitutive lysine acetylation [45]. p53^KQ/KQ^ new-born mice were smaller than their littermates, died within one day of birth, and an increased transactivation of p53 target genes was detected in their brain, liver, spleen, and thymus [45]. In this study the SET oncoprotein was shown to inhibit p53 activity by interacting with the unacetylated form of the p53 C-terminus and the acetylation of p53 C-terminal lysines prevented SET binding [45]. This latter report suggests that the increased p53 activity in unstressed p53^Δ31/Δ31^ cells might result in part from a loss of SET-mediated inhibition.

## 3. p53^Δ31/Δ31^ Mice Model Dyskeratosis Congenita, a Syndrome of Telomere Dysfunction

Although the premature death of most p53^Δ31/Δ31^ mice likely resulted from bone marrow failure and consecutive cardiac arrest, these animals also developed pulmonary fibrosis, which was identified by excessive deposits of collagen affecting the lung interstitium. This finding was particularly intriguing because, in humans, the combination of aplastic anemia and pulmonary fibrosis had been shown to characterize syndromes caused by telomere dysfunction such as dyskeratosis congenita (DC) and its severe variant the Hoyeraal-Hreidarsson syndrome (HHS) [46]. Consistent with this, shorter telomeres were observed in the bone marrow cells and MEFs from p53^Δ31/Δ31^ mice (compared to wild-type cells). Furthermore, telomere-dysfunction induced foci (TIFs), which are characterized by a co-localization of telomeric sequences with γ-H2AX signals, were much more frequent in the nuclei of p53^Δ31/Δ31^ cells [36]. 

Patients with DC or HHS carry mutations in genes encoding proteins of the telomerase complex (*DKC1*, *NHP2*, *NOP10*, *TERC*, *TERT*) or required for its assembly (*WRAP53*), components of the shelterin complex (*ACD*, *TINF2*) as well as other telomere regulators (*CTC1*, *PARN*, *RTEL1*, and possibly *NAF1* and *STN1*) [47]. However 30% to 40% of patients with DC remain unexplained at the molecular level. Mouse models knocked-out for telomerase do not develop DC-like phenotypes and exhibit telomere shortening only after several generations of intercrosses [48,49] due to a much longer initial telomere length in mice (ca. 40 kb vs. 8 kb in humans). However, a combination of mutations that affect both the telomerase and shelterin complexes such as in mTR^+/−^ Pot1b^−/−^ mice leads to telomere dysfunction and early lethality in only one generation [50]. The p53^Δ31/Δ31^ mice develop severe phenotypes of telomere syndromes and especially of DC (see Table 1 for a detailed comparison between DC features and p53^Δ31/Δ31^ mice phenotypes) in the first generation of intercrosses, which suggests that the impact of p53 activation on telomere biology is multifactorial. We demonstrated that 11 genes involved in telomere metabolism exhibit a decreased expression in p53^Δ31/Δ31^ cells compared to wild type cells [36,51]. Their expression was further decreased in response to treatment with Nutlin-3a (or Nutlin), which is an Mdm2 antagonist that specifically activates p53 [52]. Among these genes, *Dkc1* and *Gar1* encode components of the telomerase, *Tinf2* and *Terf1* encode parts of the Shelterin complex and *Rtel1* encodes a helicase involved in the replication of telomeres. Importantly, *TINF2*, *DKC1*, and *RTEL1* are mutated in a large fraction of patients with DC or HHS [53] and a *TERF1* variant has been implicated in aplastic anemia, which is a milder form of telomere syndrome [54]. Other genes implicated in telomere maintenance that we found downregulated upon p53 activation included *Blm*, *Dek*, *Fancd2*, *Fen1*, *Recql4*, and *Timeless*. The demonstration of a p53-mediated downregulation of genes essential for telomere maintenance was unexpected, but appeared physiologically important because this regulation is largely conserved in human cells [36,51]. These results revealed the importance of p53 in the regulation of telomere metabolism, which expanded the variety of functions attributed to this fascinating protein [55].

However, *TP53* germline mutations that would lead to p53 activation (e.g., nonsense mutations causing a loss of the C-terminus) were not identified in humans with DC so far. Nevertheless, it is worth noting that *PARN*, which is one of the genes mutated in DC [56], encodes a polyA ribonuclease that regulates the stability of several RNAs including the p53 mRNA [57,58] and TERC (the Telomerase RNA Component) [59]. The impact of *PARN* mutations on TERC maturation appears important because TERC overexpression was shown to increase telomere length in PARN-deficient cells [60]. However, an activation of the p53 pathway might contribute to the onset of DC features for patients carrying *PARN* mutations [56]. Furthermore, recent evidence suggests that once telomere shortening has occurred, p53 activation plays a major role in the development of hematopoietic failure in this syndrome [61].

## 4. The Fanconi Anemia DNA Repair Pathway Is Downregulated in p53^Δ31/Δ31^ Cells

More recently, our further analysis of p53^Δ31/Δ31^ cells revealed another unexpected function for p53. As mentioned previously, p53 downregulates the expression of *Fancd2*, which is a gene encoding a key protein of the Fanconi anemia (FA) DNA repair pathway [51]. The FA pathway is composed of 22 FANC proteins distributed between three complexes that induce repair of inter-strand crosslinks in order to allow the completion of DNA replication [63,64]. Missense mutations of each of the *FANC* genes have been reported to induce defects in the DNA repair pathway and lead to FA, which is another bone marrow failure syndrome (see Table 1 for detailed features of FA). Therefore, the negative regulation of *Fancd2* by p53 was very intriguing as FA is a syndrome closely related to DC. Additionally, *Rtel1*, which is one of the three genes mutated in DC and repressed by p53, encodes a Fancj-like helicase while *Blm* and *Fen1*, two other genes we found downregulated by p53, respectively, encode a helicase and an endonuclease that interact with Fanc proteins. 

These observations led us to evaluate more precisely the impact of p53 on *Fanc* gene expression. We identified 11 supplementary *Fanc* genes repressed in response to p53 hyperactivation in p53^Δ31/Δ31^ cells and in response to Nutlin. These genes encode proteins from each of the three protein complexes of the FA DNA repair pathway. We next showed that p53^Δ31/Δ31^ cells are hypersensitive to Mitomycin C (MMC), which is an inter-strand crosslink-inducing agent that leads to an increased number of chromosomal aberrations and sister chromatid exchanges. This hypersensitivity is typical of cells from FA patients [65]. Moreover, the downregulation of the FA pathway by p53 is highly conserved in human cells, which extends the potential role of p53 in the development of bone marrow failure syndromes. Similarly to what is known for DC, so far, no p53 mutation was reported to cause FA, but the p53 activation consecutive to defects in DNA repair is known to play an important role in the hematopoietic failure occurring in FA patients [66].

Furthermore, transcriptomic analyses showed that the p53 pathway is functional in low-grade ovarian serous cancers, liver cancers, and adrenocortical tumors, but are lost in high-grade carcinomas [51,67,68,69]. We found that the loss of p53 activity in these cancers correlates with an increased expression of several *FANC* genes and of other genes downregulated by p53 (e.g., *BLM*, *FEN1*, *TIMELESS*) [51]. Therefore, a concerted increase in the expression of these genes could be used as a biomarker for tumor progression. In addition we found that, for cancer cells that retain a functional p53, p53 activation by treatment with Nutlin can sensitize cells to a crosslinking agent such as MMC [51]. Since a similar synergistic effect can occur in wild-type cells [51], whether or not the therapeutic index of such an approach might be satisfactory remains to be determined.

## 5. p53-Mediated Gene Repression Often Relies on p21 and the DREAM Complex

The p53-mediated downregulation of gene expression often relies on the transactivation of p21 and the recruitment of E2F4 repressive complexes at the promoter of target genes [70,71,72,73,74,75]. Consistent with this, we showed that the p53-induced downregulation of most genes implicated in telomere metabolism and *Fanc* genes is indirect and requires p21 [36,51]. Moreover, the transcriptional repressor E2F4 is recruited at the promoter of *Rtel1* and several *Fanc* genes upon p53 activation [51].

E2F4 is a major repressive transcription factor that is a key protein of the DREAM complex (DP, RB-like, E2F4 and MuvB) [73,76]. Following p53 activation, the DREAM complex is recruited at the promoter of specific target genes in order to stop their transcription and induce cell growth arrest [73]. The p53-p21-DREAM regulatory pathway has been shown to function by recognizing specific sequences known as CDE/CHR motifs in the promoter of target genes [74]. The promoter of *Fanc* genes repressed by p53 exhibit CDE/CHR motifs required for their p53-p21-E2F4-mediated repression [51]. Mutations of the CDE part of the sequence, specifically bound by E2F4, abolish the p53-mediated downregulation of *Fanc* genes. While *Fanc* genes expression is known to vary during the cell cycle [77], their p53-dependent repression relied on CDE sequences rather than only cell cycle dynamics. 

An exception to this mechanism of p53-mediated gene repression is *Dkc1* because we found it to be downregulated by p53 independently of p21. *Dkc1* gene expression decreases upon treatment with Nutlin in both wild type cells and p21-null cells [36]. Chromatin immunoprecipitation experiments indicated that p53 binds to the *Dkc1* promoter to induce its repression. However, the underlying mechanisms for this repression are currently unknown and deserve further analysis.

## 6. p53 Regulates Genes Implicated in Centromere Structure

Our analysis of the p53^∆31^ mouse model disclosed p53^∆31/∆31^ cells as a powerful tool to identify genes repressed by the p53-p21-E2F4 regulatory pathway. Accordingly, two genes encoding proteins implicated in centromere structure were similarly shown to be downregulated in response to p53 activation [78]. The genes encoding Cenp-a, the centromeric histone-like protein, and its chaperone Hjurp exhibit decreased expression in p53^∆31/∆31^ cells. The Nutlin-induced downregulation of both genes requires p21 and CDE/CHR motifs localized in their promoters as previously described for *Fanc* genes expression. Importantly, the downregulation of *CENP-A* and *HJURP* is also conserved in human cells. In addition, this study indicated that cancer cells that have lost p53 activity become addicted to high levels of HJURP so that HJURP might be a promising therapeutic target to specifically eliminate those cells [78]. 

## 7. Biological Implications of These Results

Our analyses revealed that p53 downregulates genes required for telomere maintenance, DNA repair, and centromere structure, which is a finding that has many implications. 

### 7.1. Implications for Our Understanding of Pediatric and Developmental Syndromes

Short telomeres and defective DNA repair are known to activate p53 [79,80,81,82], but our results indicate that, conversely, increased p53 activity may affect telomere maintenance and attenuate the FA DNA repair pathway, which defines a positive regulatory feedback loop. In wild type cells, expressing a wild type p53 protein, this regulatory loop is counterbalanced by the negative regulatory loop, which results from the Mdm2-mediated degradation of p53. In contrast, in the p53^Δ31^ mouse model, the deletion of the p53 C-terminus would attenuate the negative regulation by Mdm2 leading to an abnormal hyperactivation of p53 to cause defects in telomere maintenance and DNA repair. This bipolar feedback system could explain the DC-like phenotypes developed by p53^Δ31/Δ31^ mice as well as the FA features observed in p53^Δ31/Δ31^ cells [51].

Therefore, p53^Δ31/Δ31^ cells display typical characteristics of both DC and FA, which is particularly interesting considering that these disorders share many phenotypic traits (described in Table 1) that initially led to diagnostic confusions [83,84]. Our findings suggest that sustained p53 activation might contribute to the clinical overlap between these two syndromes [51,85]. Telomeric defects have been observed in some FA patients expressing a mutation in the *FANCD2* gene [86]. Conversely, HHS patient cells mutated in *RTEL1* may also exhibit hypersensitivity to MMC [87]. In addition, FANC proteins are often associated with telomere metabolism and consolidate the link between these two cellular pathways. A recent study showed that *BRCA1* (*FANCS*) or *BRCA2* (*FANCD1*) mutations may alter the structure and function of telomeres [88]. The SNM1B (Apollo) protein is a Shelterin accessory protein, which also acts within the FA pathway [89]. FANCA is thought to participate in the co-localization of FANCD2 and TERF1 proteins to telomeres in cells that do not express telomerase [90]. FANCM, BRCA2 and BLM are necessary to resolve telomeric replication stress in cells that use alternative lengthening of telomeres (ALT) [91], and BRCA2 would allow RAD51 (FANCR) to access to telomeres in order to facilitate their replication [92]. Lastly, since FANCJ main activity is the resolution of G-quadruplex structures, some studies tend to link this protein to the maintenance of telomeres [90]. Taken together, these results strongly suggest that a better understanding of the regulation and functions of p53 may be crucial to deepen our understanding of DC and FA, and that the boundaries between these bone marrow failure syndromes need to be re-evaluated [85]. 

It is also interesting to note that patients with FA may present some of the congenital malformations found in the VACTERL-H association (Vertebral anomalies, Anal atresia, Cardiac defects, Tracheoesophageal fistula, Esophageal atresia, Renal abnormalities, Limb abnormalities, and Hydrocephalus [62]). Furthermore, another p53 mutant mouse model was recently found to pheno-copy the CHARGE syndrome (ocular Coloboma, Heart defects, choanal Atresia, Retarded growth and development, Genitourinary hypoplasia and Ear abnormalities), which is considered a VACTERL-like syndrome. In this case, a transcriptionally dead but extremely stable mutant p53 (due to multiple missense mutations in the N-terminus) was found to cause CHARGE-like features by stabilizing a wild-type p53 protein in heterozygous animals [93]. Although p53 mutations causing CHARGE were not reported in humans, the chromatin remodeler CHD7, mutated in 70% to 90% of patients with CHARGE, was shown to downregulate *TP53* gene expression. These findings illustrate that the importance of p53 in several pediatric and developmental syndromes need further investigation. 

Other mouse models displaying increased p53 activity include knocked-out mice for *Mdm2* or *Mdm4* (also known as *Mdmx*), which encode major negative regulators of p53. These mice are not viable and embryonic death is observed between 5.5 days and 11.5 days post-coitum [94,95,96,97]. Massive apoptosis was observed after the *Mdm2* loss [98] while *Mdm4* deficiency led to increased apoptosis in the brain and proliferation arrest in most other tissues [99]. p53 activation plays an important role in the death of these embryos because Mdm2 or Mdm4 deficiency is rescued by a concomitant loss of p53. Mdm2^+/−^ Mdm4^+/−^ double heterozygous mice [37] and mice expressing a hypomorphic p53 mutant over a Mdm2 null background [38] exhibit growth retardation, impaired hematopoiesis, defects in cerebellar development, and die rapidly after birth. Since these phenotypes are similar to those observed in p53^Δ31/Δ31^ animals, it would be important to re-evaluate the pathological processes occurring in these animals in the light of our results. For example, we showed that Mdm2^+/−^ Mdm4^+/−^ cells also exhibit an increased sensitivity to MMC [51]. 

### 7.2. Implications for Our Understanding of Aging Processes

Several mouse models have shown that increased p53 activity can cause accelerated aging, which is distinct from its tumor suppression capacity [11,94]. The first evidence for this came from a complex mouse model that expressed a composite mRNA encoding a truncated p53 protein that lacked 243 N-terminal residues. In p53^+/m^ mice, the heterozygous mice carrying this complex allele, the truncated p53 mutant would stabilize the wild-type p53 protein, which causes an increased resistance to cancer development. However, the mice have a surprisingly reduced lifespan correlating with features of accelerated aging [100,101]. Likewise, overexpression of p44, which is a naturally occurring shorter p53 isoform lacking 40 N-terminal residues, also led to a reduced lifespan and early aging features [102]. Recently, Hupki mice, mouse models encoding chimeric human/murine p53 genes, also provided evidence for the importance of p53 in regulating aging and longevity. In humans, a SNP encoding either an arginine (R72) or a proline (P72) at codon 72 is known to influence p53 function with the P72 allele associated with weaker p53 activity and tumor suppression capacity. Hupki mice carrying the P72 SNP exhibited higher cancer risk, but had a delayed development of aging-associated phenotypes [103]. A patient displaying early-aging features was recently shown to carry an *MDM2* mutation reducing its capacity to inhibit p53 [104]. Although these studies provide compelling evidence for an impact of p53 activation on aging processes, the underlying mechanisms remain to be fully understood. 

In a landmark review, López-Otín et al [105] proposed nine hallmarks of aging grouped into three categories: (1) four primary hallmarks would cause cellular damage including genomic instability, telomere attrition, epigenetic alterations, and loss of proteostasis; (2) as a response to these primary damages, three antagonistic hallmarks would initially mitigate the damage but eventually become deleterious—these would include altered nutrient sensing, mitochondrial dysfunction, and cellular senescence; (3) integrative hallmarks, i.e., the end results of the previous hallmarks, would be responsible for functional decline—these would be stem cell exhaustion and altered intercellular communication. Accordingly, a simplified model for aging caused by telomere attrition would be the following: (1) in human differentiated cells, since the DNA replication machinery is unable to fully duplicate the end of linear chromosomes, telomeres would shorten with each cell division; (2) once telomeres become critically short, they trigger a DNA damage response that activates p53, which then transactivates *CDKN1A* and *PAI-1* to induce cellular senescence or represses *PGC1α* and *PGC1β* to impair mitochondrial biogenesis and function, which would promote cell death; (3) increased cell death or the clearance of senescent cells would mobilize stem cells to re-establish cell numbers, which eventually leads to stem cell exhaustion [105]. Our results suggest that the impact of p53 activation on aging processes is not limited to a secondary triggering of cellular responses inducing mitochondrial dysfunction or senescence. Rather, by showing that p53 can downregulate genes required for telomere maintenance and that increased p53 activity leads to short and dysfunctional telomeres, we provided evidence that p53 activation can cause telomere attrition, which is a primary hallmark of aging.

### 7.3. Implications for Our Understanding of How p53 May Act as a Guardian of the Genome

At first glance, finding that p53 downregulates genes important for telomere maintenance, DNA repair, and centromere structure seems counter-intuitive since it appears to contradict the concept of p53 as a “guardian of the genome.” However, our results are supported by other independent studies. In a series of reports combining bioinformatics meta-analyses and transfection approaches, Engeland and colleagues concluded that more than 250 genes are indirectly downregulated by p53 in a p21/DREAM-dependent manner. These genes belong to groups of functionally-related genes that control many checkpoints of the cell cycle including genes involved in DNA repair, centromere organization, and telomere maintenance [76]. Furthermore, we showed that p53 downregulates the FA DNA repair pathway so that, after 48 hours of treatment with Mitomycin C, an increased p53 activity correlates with an increased frequency of chromosomal rearrangements [51]. Consistent with this, a recent study showed that the concomitant activation of p53 and inhibition of CDK/cyclin complexes in normal human cells leads to a premature senescence that correlates with a decreased expression of DNA repair genes and with the accumulation of DNA damage [106]. It seems complex to reconcile these recent data with the concept of “guardian of the genome” proposed by David Lane more than 25 years ago [30]. 

**Figure 1:**
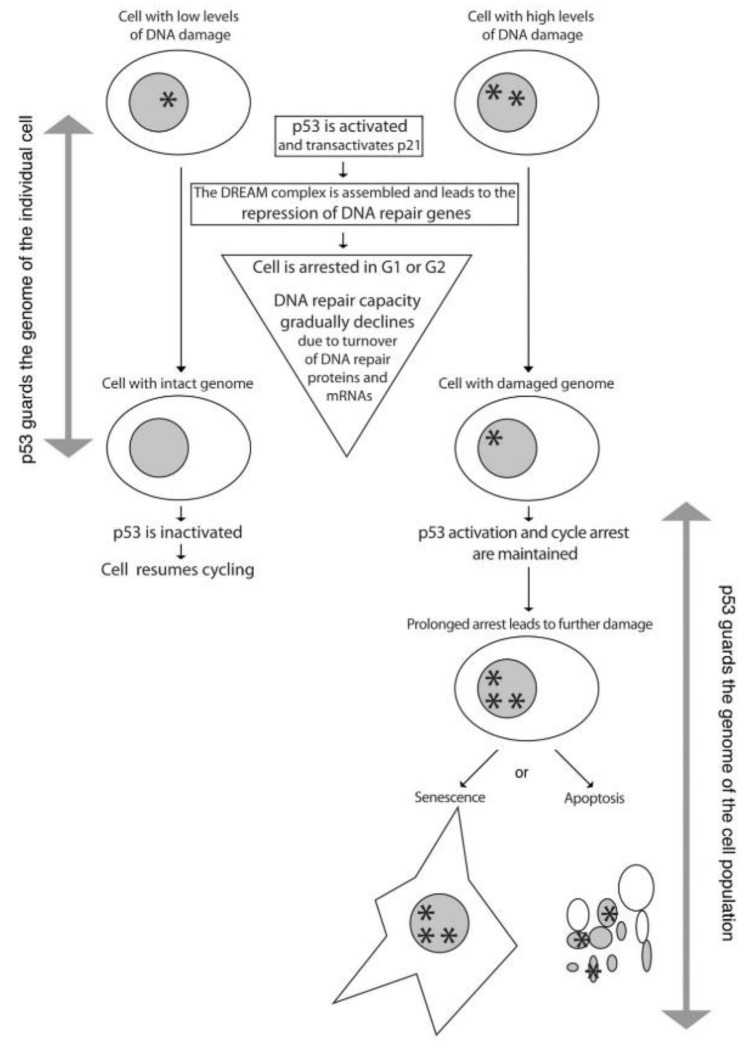
The “guardian of the genome” model revisited. This is a simplified model designed to directly discuss the “guardian of the genome” model initially proposed by David Lane [30]. Therefore, we consider here that p53 responds to DNA damage and induces a G1 or G2 arrest that may or may not lead to senescence or apoptosis. In addition to this, we now know that p53 responds to a large variety of cellular stresses and promotes many different cellular responses [107] and that it might be differently regulated in some tissues or in tumor cells to favor a pro-apoptotic response [108].

In his model, David Lane proposed that if DNA is damaged, p53 would accumulate and lead to G1 arrest to allow extra-time for repair before division, but if the repair fails, p53 may trigger cell suicide by apoptosis [30]. To reconcile the “guardian of the genome” model with the data that correlate p53 activation with increased DNA damage, it is perhaps necessary to consider the kinetics of the system. Nutlin was shown to lead to an efficient downregulation of DNA repair genes in 24 hours [51,106], but evidence of increased DNA damage was observed after 48 hours [51] or 72 hours [106] of Nutlin. Presumably, the effect of a downregulation of DNA repair genes would depend on the turnover of DNA repair proteins (and mRNAs encoding them) present in the cell before the arrest so that there might be a short time window in which arrested cells can repair DNA lesions efficiently. If repair occurs, they would resume cycling with an intact genome. However, once this time window has passed, the prolonged effect of p53 activation would operate, i.e., a decreased capacity to repair DNA lesions, and lead to further DNA damage that might seal the fate of damaged cells towards apoptosis or senescence. As shown in Figure 1, according to this updated model, p53 might act in the short-term as a guardian of the genome of the individual cell, while in the longer-term it would rather act as a guardian of the genome of the cell population.

## 8. Conclusions

Our finding that p53 downregulates genes required for genome maintenance initially came as a surprise given the well-accepted notion that p53 acts as “the guardian of the genome.” On second thought, however, the downregulation of these genes may actually contribute to the toolkit used by p53 to prevent tumor formation. Furthermore, because some of the genes downregulated by p53 are important for hematopoiesis or the aging processes, the implications of our results go beyond cancer research. In the future, it will be particularly important to determine to what extent our findings, obtained from studying a mouse model, are relevant to human health and disease.

## Figures and Tables

**Table 1:** Phenotypical traits of dyskeratosis congenita and Fanconi anemia and their observation in p53^Δ31/Δ31^ mice. The sustained p53 activation displayed in the mutant mice leads to the development of features typical of both bone marrow failure syndromes [36,51].

Syndrome	Type of Feature	Phenotypes	p53^Δ31/Δ31^ Mice
**Dyskeratosis congenita**	Specific features of diagnostic	Very short telomeres; reticular skin pigmentation; nail dysplasia; oral leucoplakia	√
	Pathological traits	Pancytopenia; bone marrow failure; pulmonary fibrosis; short stature; cardiac hypertrophy	√
	Hoyeraal Hreidarsson syndrome specificity	Cerebellar hypoplasia; immunodeficiency; developmental delay	Cerebellar hypoplasia
	Associated features	Liver or gastrointestinal disease; premature grey hair; avascular necrosis of the hips; microcephaly; testicular atrophy	Testicular atrophy
	Predisposition to cancer development	Leukemia; squamous cell cancers of head, neck, and anogenital region; myelodysplastic syndromes	Not observable *
	Impaired molecular mechanism	Telomere maintenance	√
**Fanconi anemia**	Specific features of diagnostic	Increased chromosomal abnormalities in clastogenic assay and progressive bone marrow failure	√
	Pathological traits	Pancytopenia; short stature; skin abnormalities (“café-au-lait” macules, hyper-/hypo-pigmented spots)	√
	Associated features	Upper limb abnormalities; microcephaly; microphthalmia; triangular “Fanconi” face; renal and cardiac anomalies; testicular atrophy; may have features of VACTERL-H ** association	Testicular atrophy Microphtalmia ***
	Predisposition to cancer development	Leukemia; squamous cell cancers of head, neck, and anogenital region; skin and digestive tract carcinomas; mammary gland and ovary tumor; brain tumor; myelodysplastic syndromes	Not observable *
	Impaired molecular mechanism	Fanconi anemia DNA repair pathway	√

* The p53^Δ31/Δ31^ mice die prematurely generally around four weeks after birth, which prevents the ascertainment of tumor development. ** VACTERL-H: Vertebral anomalies, Anal atresia, Cardiac defects, Tracheoesophageal fistula, Esophageal atresia, Renal abnormalities, Limb abnormalities, and Hydrocephalus [62]. *** Rarely observed.
